# Selective Enrichment of Angiomirs in Extracellular Vesicles Released from Ischemic Skeletal Muscles: Potential Role in Angiogenesis and Neovascularization

**DOI:** 10.3390/cells13151243

**Published:** 2024-07-24

**Authors:** Sylvie Dussault, Michel Desjarlais, Nozha Raguema, Eric Boilard, Sylvain Chemtob, Alain Rivard

**Affiliations:** 1 Department of Medicine, Centre Hospitalier de l’Université de Montréal (CHUM) Research Center, Montréal, QC H2X 0A9, Canada; sylvie.dussault.chum@ssss.gouv.qc.ca (S.D.); nozha.raguema.chum@ssss.gouv.qc.ca (N.R.); 2 Departments of Pediatrics, Ophthalmology and Pharmacology, Centre Hospitalier Universitaire Sainte-Justine Research Center, Montréal, QC H3T 1C5, Canada; michel.desjarlais@umontreal.ca (M.D.); sylvain.chemtob@umontreal.ca (S.C.); 3 Department of Infectious Diseases and Immunity, Centre de Recherche du Centre Hospitalier Universitaire de Québec, Université Laval, Québec City, QC G1V 0A6, Canada; eric.boilard@crchudequebec.ulaval.ca

**Keywords:** extracellular vesicles, microvesicles, exosomes, microRNA (miR), ischemia, angiogenesis, neovascularization

## Abstract

MicroRNAs (miRs) regulate physiological and pathological processes, including ischemia-induced angiogenesis and neovascularization. They can be transferred between cells by extracellular vesicles (EVs). However, the specific miRs that are packaged in EVs released from skeletal muscles, and how this process is modulated by ischemia, remain to be determined. We used a mouse model of hindlimb ischemia and next generation sequencing (NGS) to perform a complete profiling of miR expression and determine the effect of ischemia in skeletal muscles, and in EVs of different sizes (microvesicles (MVs) and exosomes) released from these muscles. Ischemia significantly modulated miR expression in whole muscles and EVs, increasing the levels of several miRs that can have pro-angiogenic effects (angiomiRs). We found that specific angiomiRs are selectively enriched in MVs and/or exosomes in response to ischemia. In silico approaches indicate that these miRs modulate pathways that play key roles in angiogenesis and neovascularization, including HIF1/VEGF signaling, regulation of actin cytoskeleton and focal adhesion, NOTCH, PI3K/AKT, RAS/MAPK, JAK/STAT, TGFb/SMAD signaling and the NO/cGMP/PKG pathway. Thus, we show for the first time that angiomiRs are selectively enriched in MVs and exosomes released from ischemic muscles. These angiomiRs could be targeted in order to improve the angiogenic function of EVs for potential novel therapeutic applications in patients with severe ischemic vascular diseases.

## 1. Introduction

Cardiovascular diseases (CVDs) are a leading cause of mortality and morbidity worldwide. Among CVDs, peripheral artery disease (PAD) is a major health concern, affecting more than 200 million men and women worldwide and being the third-leading cause of cardiovascular morbidity after coronary artery disease and stroke [1]. PAD is associated with skeletal muscle pain during activity (claudication) and impaired mobility. In its more severe forms, PAD leads to chronic ischemia with rest pain, non-healing ulcers, tissue necrosis and gangrene leading to lower limb amputation. Frequently, the atherosclerotic process is so diffuse that revascularization techniques by angioplasty or bypass surgery cannot be performed. Therefore, enhancing the physiological capacity of the organism to develop new blood vessels (neovascularization) could be an attractive strategy to improve perfusion of affected muscles, reduce pain and avoid ischemic tissue damage and limb amputation [2,3].

Neovascularization involves the development of collateral vessels (arteriogenesis) as well as the activation, proliferation and migration of mature endothelial cells that will extend the pre-existing vascular network (angiogenesis) [4]. The angiogenic process is modulated by the microenvironment and by different growth factors. Vascular endothelial growth factor (VEGF) has been shown to have a critical role in the induction of angiogenesis [5]. Under hypoxic conditions, VEGF is induced via hypoxia-inducible factor 1 (HIF1) and acts on ECs to induce vascular permeability, EC proliferation, cellular migration and tubule formation [6]. VEGF signaling is associated with p44/42 MAPK and p38 MAPK activation, for EC proliferation and migration, respectively, and PI-3 kinase/Akt/NO pathway induction for EC survival and migration [7,8]. Although the specific mechanisms that govern these different signals are not completely understood, it is increasingly recognized that microRNAs (miRNAs or miRs) are involved in the modulation of the angiogenic process.

miRs represent a novel class of endogenous non-coding small RNA molecules (20–25 nucleotides) that regulate a wide range of physiological and pathological processes, including angiogenesis [9,10,11]. They can suppress protein synthesis by inhibiting the translation of mRNA to protein or by promoting mRNA degradation, thereby silencing gene expression. Although the postnatal function of individual miR in vivo under pathological conditions remains largely undefined, specific miRs have been shown to modulate angiogenesis in different contexts [11]. It has also been proposed that forced expression of angiomiRs could represent a novel therapeutic strategy to improve ischemia-induced neovascularization in atherosclerotic conditions [12,13,14]. However, before this type of therapeutic approach can be developed, it is critical to understand how angiomiRs are modulated, packaged and transferred between cells in physiological and pathological conditions.

Cells can release different types of extracellular vesicles (EVs) including exosomes released from intracellular compartments (~30–120 nm in diameter) and microvesicles released by plasma membrane budding (MVs, ~100–1000 nm in diameter). EVs have emerged as important players for cell-to-cell communication in normal physiology and in pathological conditions [15,16,17]. Released EVs may interact with the originator cells, therefore acting as autocrine mediators, and with other cell types, thus acting as paracrine mediators [16,18]. EVs can carry a variety of molecular cargo including nucleic acids, lipids, proteins and metabolites. The miR content of EVs and the transfer of pro-angiogenic miRs to receptive cells seem particularly important for the stimulation of angiogenesis [19]. However, and especially in the context of ischemic vascular diseases, the specific miRs that are packaged in EVs released locally from skeletal muscles, and how this process is modulated by ischemia, remain to be determined. Here, in a mouse model of PAD, we used next-generation sequencing (NGS) to perform a complete profiling of miR expression and determine the effect of ischemia in skeletal muscles, and in EVs of different sizes (MVs and exosomes) released from these muscles. Our results indicate that several miRs with angiogenic potential are selectively enriched in MVs and exosomes produced in ischemic muscles. These angiomiRs are predicted to modulate key pathways involved in angiogenesis and neovascularization, and could potentially be targeted in order to improve the angiogenic function of EVs and promote therapeutic neovascularization in PAD patients with critical limb ischemia.

## 2. Materials and Methods

**Murine hindlimb ischemia.** The protocol was approved by the Comité Institutionnel de Protection des Animaux (CIPA) of the Centre Hospitalier de l’Université de Montréal (CHUM). Unilateral hindlimb ischemia was surgically induced in 6- to 8-week-old C57BL/6 mice by removing the femoral artery under anesthesia with 2% isoflurane [13]. Ischemic and non-ischemic muscles were harvested at 1, 2, 4 and 7 days post-ischemia.

**Electronic microscopy.** Ischemic muscles were removed and fixed with 2.5% glutaraldehyde/4% paraformaldehyde in 0.1 M sodium phosphate buffer (PB) pH 7.4. Samples were washed in PB, followed by a post-fixation with 1% OsO_4_ in PB, for an hour at 4 °C. Tissue samples were then dehydrated in graded series of ethanol and embedded in Epon resin. Ultrathin sections were obtained using a Reichert Ultracut ultramicrotome and mounted on naked nickel grids. Sections were stained with uranyl acetate and lead citrate and examination was performed using a Philips CM100 transmission electron microscope (Philips, Eindhoven, The Netherlands) operated at 80 kV. Electron micrographs were captured with an AMT XR80 digital camera.

**Extracellular vesicles isolation.** Thigh and calf muscles were rinsed in sterile PBS (Wisent, St-Jean-Baptiste, QC, Canada) and minced thoroughly for 5 min with fine scissors into 37 mL of DMEM (Wisent). The mixture was then centrifuged at 3000× *g* for 3 min. The supernatant was then passed through a cell strainer (70 µm) and centrifuged again at 3000× *g* for 3 min to remove cells and debris. The supernatant was recentrifuged at 20,500× *g* for 45 min to pellet MVs. This supernatant was then recentrifuged at 120,000× *g* for 3 h to pellet exosomes.

**Cytometry.** Vesicles were stained for 30 min with 5µM Carboxyfluorescein succinimidyl ester (CFSE, Thermo Fisher, Waltham, MA, USA). All samples were treated with 0.5% NP40 detergent to dissolve CFSE+ vesicles and control for protein aggregates. Flow cytometry analyses were performed on a SORP BD FACSAria III (BD Biosciences, San Jose, CA, USA) equipped with a 100 mW 488 nm laser, a 50 mW 405 nm laser and a PMT for forward-scattered light (FSC) detection of the 488 nm laser. The side-scattered light (SSC) was detected from the 405 nm laser for excitation and a 405/10 nm bandpass filter for emission detection. Samples were acquired at low sample pressure with a 100-micron nozzle, sheath fluid (BD FACS Sheath fluid; BD Biosciences) pressurized at 20 PSI (pounds per square inch) and no neutral density filter. The instrument cleaning procedure prior to acquisition was the following: 30 min 10% bleach, 30 min BD Detergent solution (BD Biosciences). The instrument was then stabilized by running 100 nm-filtered molecular grade sterile DNase/RNase-free water for 1 h to minimize instrument background noise prior measuring samples. Acquisition was carried out with BD FACSDIVA version 8.0.1, and CS&T was run prior each experiment to control for appropriate laser delays. CFSE+ vesicles containing samples were measured over 120 s at the slowest possible flow rate (10 μL/min) and each sample was recorded in duplicate to control for the stability of the instrument. Flow cytometry data were analyzed using fluorescence (CFSE+) and PMT collected forward-scattered light (FSC) parameters in height. The pulse height (-H) as the intensity of the signal is the most accurate parameter over the pulse area parameter (-A) for analysis of submicron particles: the area integrates the value of pulse height and the pulse width (time-of-flight, -W). However, the time-of-flight measurements of small particles is less precise leaving the pulse height as the most accurate parameter for the analysis.

**RNA Extraction and Sequencing.** Total RNA was extracted from muscles using the Ambion mirVana miRNA Isolation Kit (ThermoFisher Scientific, Waltham, MA, USA) according to the manufacturer’s protocol. Total RNA from EVs was extracted with the miRNeasy mini kit (Qiagen, Hamburg, Germany). RNA was validated on a Bioanalyzer (Agilent, Santa Clara, CA, USA) using an RNA Pico chip. Libraries were prepared using a QIAseq miRNA Library Kit (Qiagen). Sequencing was performed on a NextSeq 500 (Illumina, San Diego, CA, USA), obtaining around 5 million reads per sample. The QIAseq miRNA sequencing files were uploaded to the GeneGlobe Data Analysis center. Normalization by library size was performed, and values expressed as Unique Molecular Index (UMI). miRs were considered to be upregulated or downregulated if the fold-change value was greater than 1.2 for upregulated miRs and lower than 0.8 for downregulated miRs.

**Bioinformatic analysis.** miR and target gene sequences were extracted from the mirdb database as previously described [20,21]. Heatmaps were generated by the heatmapper website [22]. Pathway unions and gene unions were generated with miRPathv4 [23].

**Statistical analysis.** For sequencing results, the GeneGlobe Analysis Center (Qiagen) provided a calculated *p*-value based on the Wald test for RNA fold changes. Cytometry results for MV numbers at day 2 after ischemia are presented as Mean ± SEM, statistical significance was evaluated by a *t*-test and *p* < 0.05 was used to denote statistical significance.

## 3. Results

### 3.1. miR Expression Profile in Whole Skeletal Muscles and Modulation by Ischemia

**Figure 1:**
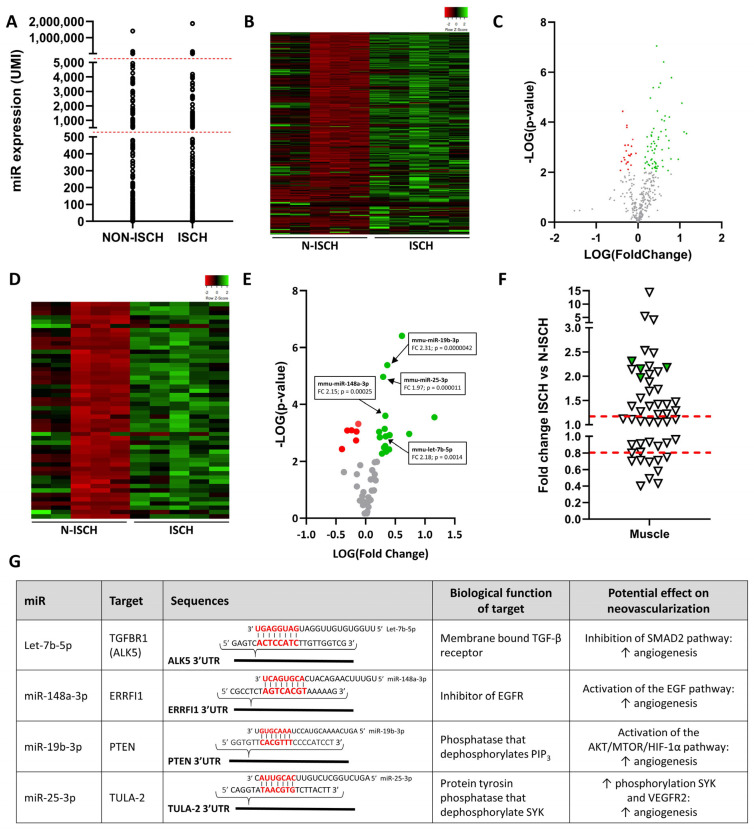
**Effect of ischemia on miR expression profile in skeletal muscles.** (**A**,**B**) General distribution (**A**) and heatmap representation (**B**) of miR expression in non-ischemic (N-ISCH) and ischemic (ISCH) skeletal muscles. (**C**) Volcano representation of the effect of ischemia on miR expression in skeletal muscles. miRs significantly decreased or increased are shown in red or green, respectively. (**D**,**E**) Heatmap (**D**) and volcano (**E**) representations of the effect of ischemia on the 50 most expressed miRs in ischemic muscles. miRs significantly decreased or increased are shown in red or green, respectively. Four potential angiomiRs were identified (arrows). (**F**) Fold change representation of the effect of ischemia on the 50 most expressed miRs in ischemic muscles. Potential angiomiRs identified are shown in green. (**G**) Targets, biological function and angiogenic effects of angiomiRs significantly increased by ischemia in skeletal muscles. FC = fold change. N = 4/group.

We used next generation sequencing (NGS) to perform a complete profiling of miR expression in whole skeletal muscles at baseline (normoxia), and 2 days after the induction of hindlimb ischemia. A total of 370 different miRs were detected in skeletal muscles (Figure 1). Although the general distribution of miR expression was similar in non-ischemic and ischemic muscles (Figure 1A), heatmap representation shows that ischemia leads to important modulation in the expression level of various miRs (Figure 1B). As shown on the volcano plot in Figure 1C, several miRs are significantly modulated after ischemia, more miRs being increased (green) vs. decreased (red) in ischemic conditions. However, several of these modulated miRs have very low expression (i.e., <100 UMI) and therefore their physiological impact might be limited. Focusing on the 50 most-expressed miRs in ischemic muscles, heatmap representation confirms that more miRs are increased vs. decreased after ischemia (Figure 1D). Among the modulated miRs, we identified four potential angiomiRs that are significantly increased by ischemia (Figure 1E–G). Let-7b-5p promotes angiogenesis in vitro and in vivo through targeting of the antiangiogenic TGFBR1 [24]. miR-148a-3p targets ERRFI1, an inhibitor of EGF receptor (EGFR), which leads to activation of the pro-angiogenic EGF pathway [25]. miR-19b-3p targets PTEN, leading to preservation of PIP3 phosphorylation and activation of the AKT/mTOR/HIF-1α angiogenic pathway [26]. Finally, miR-25-3p targets TULA-2, preserving SYK phosphorylation and inducing VEGFR2-dependent angiogenesis [27] (Figure 1G).

### 3.2. Effect of Ischemia on Skeletal Muscle EV Number and miR Cargo

**Figure 2:**
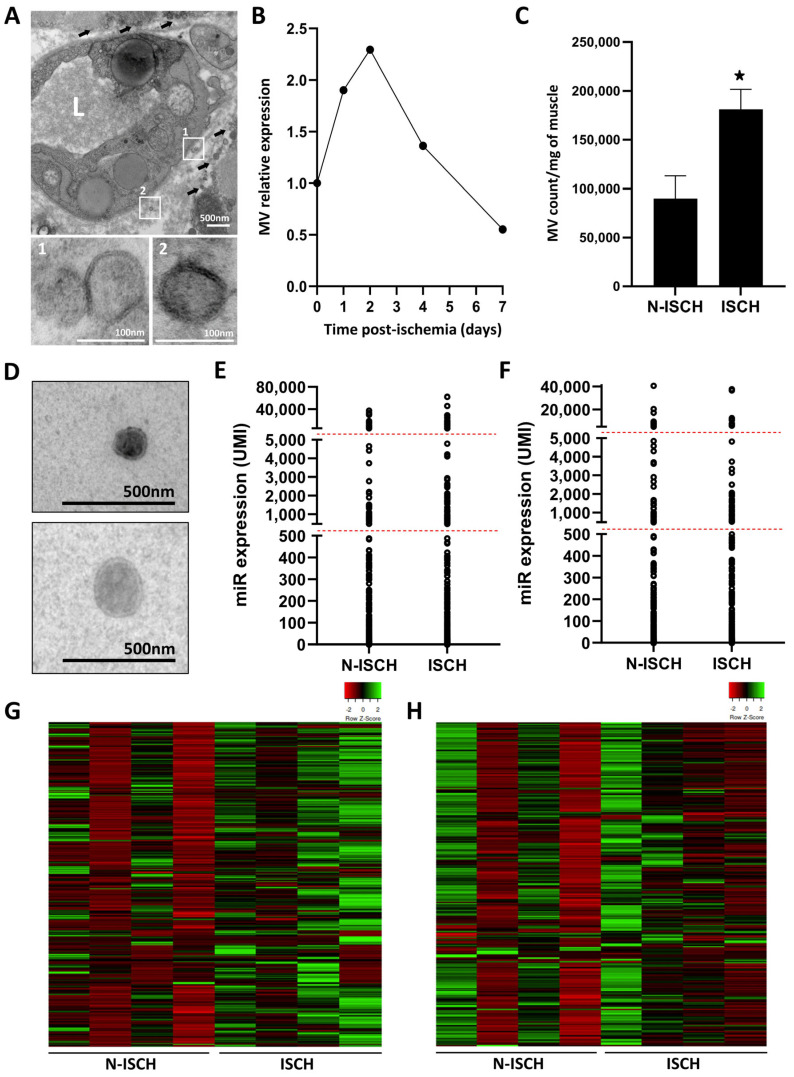
**miR profile and effect of ischemia in extracellular vesicles (EVs) isolated from skeletal muscles.** (**A**) Electron microscopy images with higher magnifications (white squares 1 and 2) of EVs near capillaries in ischemic muscles. Black arrows indicate vesicles of different sizes. L = lumen. (**B**,**C**) Time course of MV release after ischemia (**B**) and comparison of MV counts in ischemic vs. non-ischemic muscles two days after surgery (**C**), as assessed by flow cytometry. * *p* ˂ 0.05 vs. N-ISCH. (**D**) Examples of MVs isolated from skeletal muscles by centrifugation. (**E**–**H**) General distribution and heatmap representation of miR expression in microvesicles (**E**,**G**) and exosomes (**F**,**H**) isolated from non-ischemic (N-ISCH) and ischemic (ISCH) skeletal muscles two days after surgery. N = 4/group.

**Figure 3:**
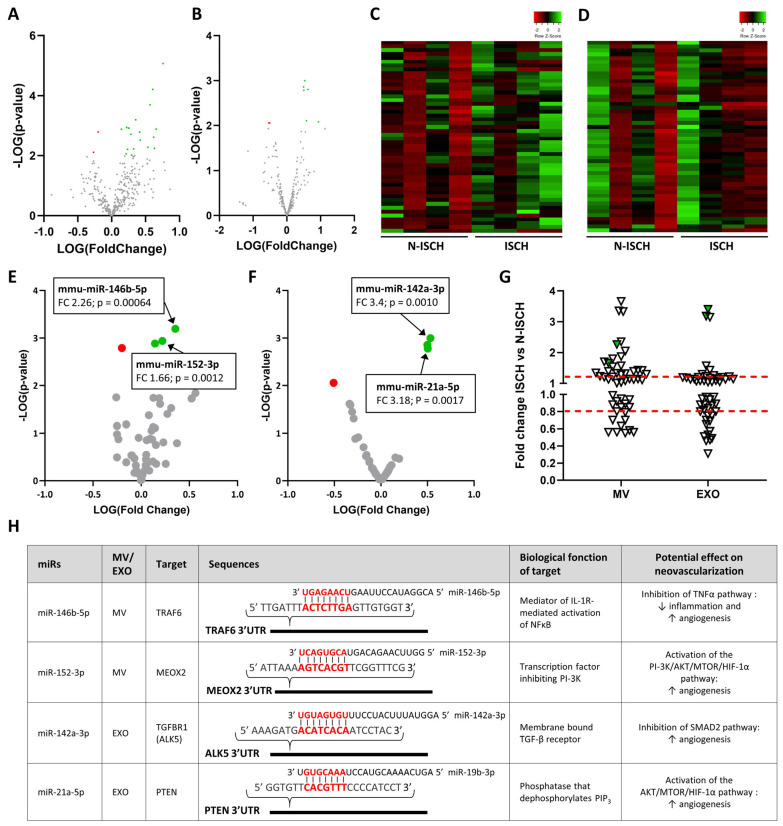
**Identification of AngiomiRs in EVs released from ischemic skeletal muscles.** (**A**,**B**) Volcano representation of the effect of ischemia on general miR expression in microvesicles (**A**) and exosomes (**B**). (**C**–**F**) Heatmap and volcano representations of the effect of ischemia on the 50 most expressed miRs in ischemic microvesicles (**C**,**E**) or exosomes (**D**,**F**). miRs significantly decreased or increased are shown in red or green, respectively. (**G**) Fold-change representation of the effect of ischemia on the 50 most expressed miRs in microvesicles (MV) and exosomes (EXO). Potential angiomiRs identified are shown in green. (**H**) Description of the targets, biological function and angiogenic effects of angiomiRs that are significantly increased by ischemia in microvesicles (MV) and exosomes (EXO). FC = fold change. N = 4/group.

miRs can be transferred between cells by extracellular vesicles, including MVs and exosomes, and these vesicles have the ability to act as paracrine mediators in tissues. Using electron microscopy imaging in skeletal muscles, we could identify several vesicles of different sizes that exhibit a bilayer membrane (Figure 2A,D). Quantification of CFSE+ vesicles by cytometry shows that the number of MVs significantly increases after ischemia in skeletal muscles, with a maximal effect observed two days after surgery (Figure 2B,C). We then performed a complete profiling of the miR cargo of MVs and exosomes released in skeletal muscles. A total of 355 and 354 miRs were detected in MVs and exosomes, respectively. As shown on Figure 2E,F, the overall distribution of miRs is similar in MVs and exosomes. However, heatmap representation shows that ischemia leads to important modulation in the expression level of various miRs, both in MVs (Figure 2G) and in exosomes (Figure 2H). As shown on the volcano plot in Figure 3A,B, several miRs are significantly modulated after ischemia, more miRs being increased (green) vs. decreased (red) in ischemic conditions. Focusing on the 50 most expressed miRs in ischemic EVs, we identified four miRs (two in MVs and two in exosomes) that are significantly increased by ischemia and that could act as angiomiRs (Figure 3C–G). In MVs, miR-146b-5p is known to target TRAF6, inhibiting TNF-dependent inflammation and increasing physiological angiogenesis [14]. miR-152-3p targets mesenchyme homeobox 2 (MEOX2) to maintain the angiogenic activities of endothelial cells [28]. In exosomes, miR-142a-3p targets the TGFBR1 ALK5, leading to inhibition of SMAD2 pathway and improved angiogenesis [29]. miR-21a-5p targets PTEN, leading to preservation of PIP3 phosphorylation and activation of the AKT/mTOR/HIF-1α angiogenic pathway [30] (Figure 3G).

### 3.3. Hierarchical Ranking of miR Expression in Whole Ischemic Muscles, MVs and Exosomes

**Figure 4:**
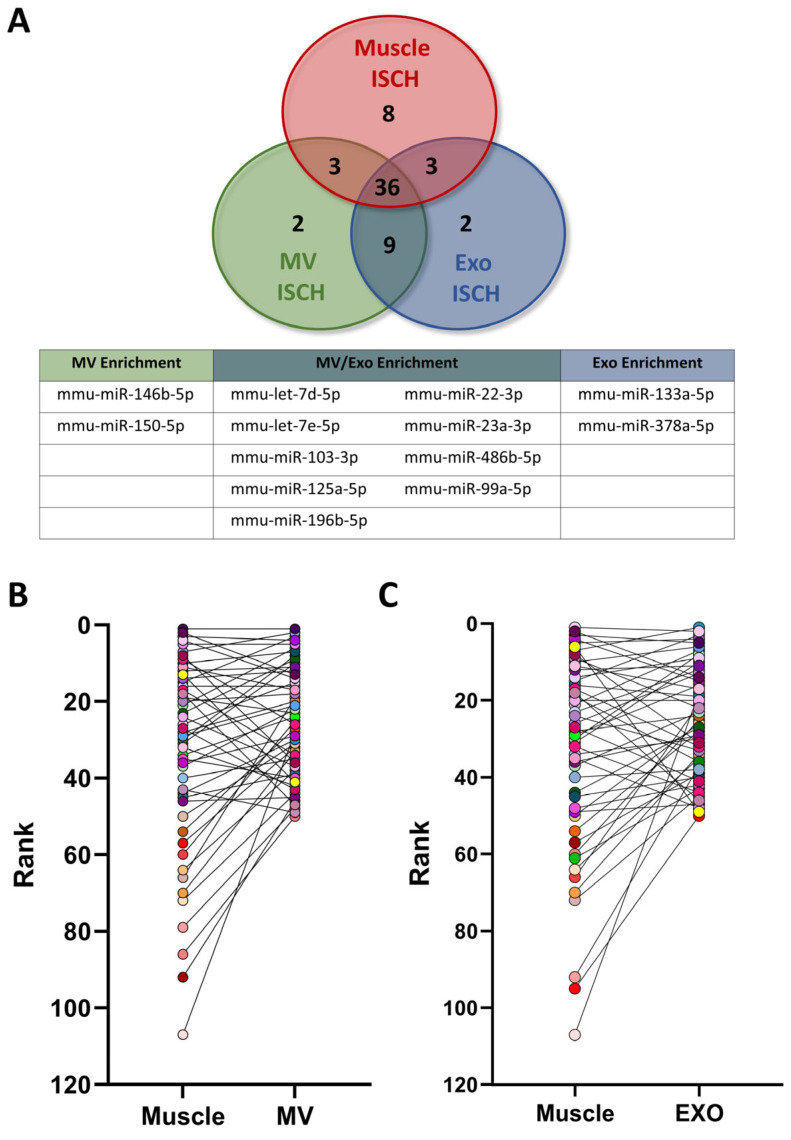
**Hierarchical ranking of miR expression in skeletal muscles and EVs in ischemic conditions.** (**A**) Venn diagram comparing the 50 most expressed miRs in whole skeletal muscles, MVs and exosomes in ischemic conditions. The table shows miRs that are enriched in microvesicles (MV), exosomes (EXO), or both, compared to whole skeletal muscles. (**B**,**C**) Leap in hierarchical ranking of the 50 most expressed miRs in microvesicles (**B**) and exosomes (**C**) compared to skeletal muscles in ischemic conditions. N = 4/group.

The effect of ischemia on the packaging of miRs in EVs released from skeletal muscles is unknown. We hypothesized that ischemia could lead to selective enhancement of angiomiRs in EVs. We identified and compared the 50 most expressed miRs in whole skeletal muscles, MVs and exosomes in ischemic conditions (Figure 4A). We found that 36 highly expressed miRs are common to whole skeletal muscles, MVs and exosomes. However, in ischemic conditions, nine miRs are enriched in EVs (both in MVs and exosomes) whereas two miRs are specifically enriched in MVs, and two other miRs are specifically enriched in exosomes (Figure 4A).

**Figure 5:**
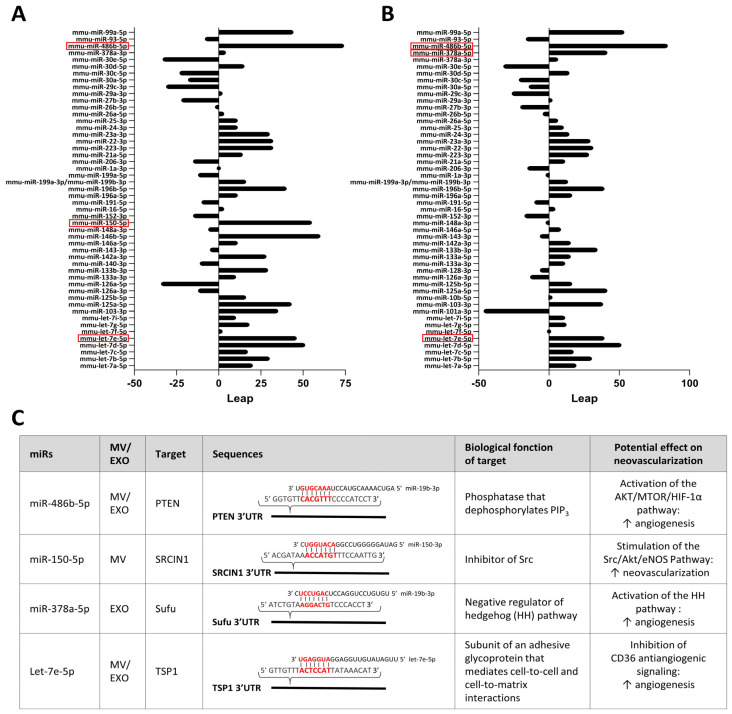
**Identification of angiomiRs enriched in EVs released from ischemic skeletal muscles.** (**A**,**B**) Quantitative representation of the leap in hierarchical ranking of the 50 most expressed miRs in microvesicles (**A**) and exosomes (**B**) compared to skeletal muscles in ischemic conditions. Potential angiomiRs identified are highlighted in red. (**C**) Description of the targets, biological function and angiogenic effects of angiomiRs that are enriched in microvesicles (MV) and exosomes (EXO) following ischemia. N = 4/group.

We then analyzed the leap in hierarchical ranking when looking at the expression of miRs in whole ischemic skeletal muscles compared to MVs and exosomes released in ischemic muscles (Figure 4B,C and Figure 5A,B). Among the miRs demonstrating the most important increase in hierarchical ranking in EVs (Figure 5A,B), we identified four miRs with angiogenic potential that are enriched in MVs and/or exosomes (Figure 5C). miR-486b-5p is enriched in both MVs and exosomes and targets PTEN, leading to preservation of PIP3 phosphorylation and activation of the AKT/mTOR/HIF-1α angiogenic pathway [31]. Let-7e-5p is also enriched in both MVs and exosomes. It targets TSP1 leading to inhibition of CD36 antiangiogenic signaling [32]. miR-150-5p is specifically enriched in MVs. It targets SRCIN1, leading to stimulation of the angiogenic Src/Akt/eNOS pathway [13]. miR-378a-5p on the other hand is specifically enriched in exosomes. It targets Sufu, leading to stimulation of the hedgehog (HH) pathway, which increases angiogenesis [33] (Figure 5C).

### 3.4. Predictive Pathways Modulated by Enriched miRs in Ischemic Muscles, MVs and Exosomes

**Figure 6:**
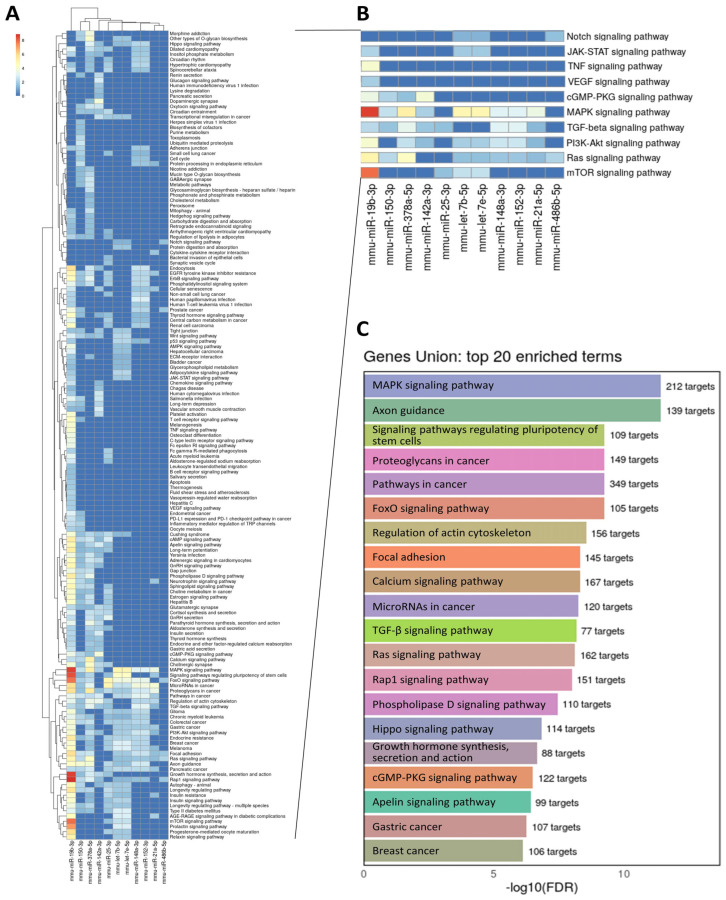
**Predictive pathways modulated by enriched miRs in ischemic muscles and EVs.** KEGG pathway unions (**A**,**B**) and gene unions (**C**) identified by miRPathv4 and predicted to be modulated by miRs that are increased in ischemic skeletal muscles, upregulated by ischemia in EVs (MVs and exosomes) or selectively enriched in EVs in response to ischemia. N = 4/group.

**Figure 7:**
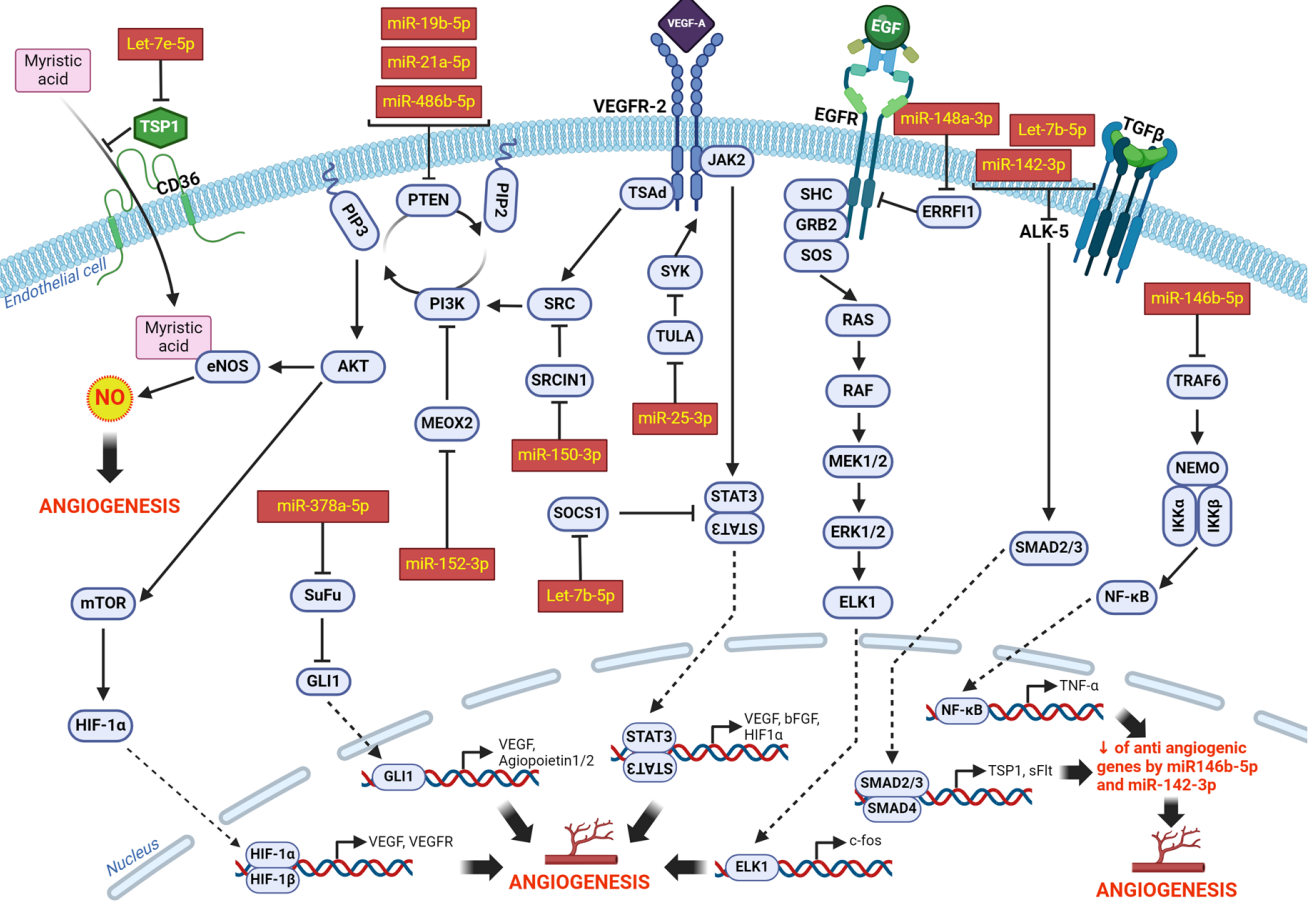
**Integrated model of the angiogenic effect of miRs that are enriched in muscles and EVs in ischemic conditions.** Summary of the role of angiomiRs that are induced in ischemic skeletal muscles and/or selectively enriched in EVs following ischemia, and that could synergistically combine their physiological function to promote angiogenesis and neovascularization through the activation of multiple pathways.

Our study identified several potential angiomiRs that are either significantly increased in ischemic skeletal muscles (Let-7b-5p, miR-148a-3p, miR-19b-3p, miR-25-3p), upregulated by ischemia in EVs (miR-146b-5p, miR-152-3p, miR-142a-3p, miR-21a-5p) or selectively enriched in EVs in response to ischemia (miR-486b-5p, Let-7e-5p, miR-150-5p, miR-378a-5p). We studied these different miRs as a group and used miRPathv4 to perform prediction of functional miR targets, pathway unions and gene unions that are affected by those miRs. As seen on Figure 6A,B, collectively these miRs are predicted to modulate several key pathways that are involved in angiogenesis including VEGF, MAPK, cGMP/PKG and PI3K/Akt. Gene unions (Figure 6C) confirm that the identified miRs modulate a high number of targets involved in processes that are related to angiogenesis including cancer, stem cell pluripotency, MAPK, Ras and cGMP/PKG signaling pathways. Overall, our results suggest that angiomiRs, induced in ischemic skeletal muscles and/or selectively enriched in EVs following ischemia, could synergistically combined their physiological function to promote angiogenesis and neovascularization through the activation of multiple pathways (Figure 7).

## 4. Discussion

To our knowledge, this is the first comprehensive description of the effect of ischemia on miR expression profiling both in skeletal muscles and in EVs that are produced locally in the context of peripheral artery diseases (PAD). It is also the first study comparing miR cargos in EVs of different sizes, i.e., exosomes and MVs, in the setting of limb ischemia. EVs have previously been shown to promote angiogenesis and neovascularization, and one of the main mechanisms involved in the stimulation of angiogenesis by EVs has been attributed to the transfer of pro-angiogenic miRs [19]. However, little is known about the precise role of EVs in the context of ischemic vascular diseases. Most studies so far have used EVs isolated from the peripheral blood, often with the goal of identifying miRs that could be used as biomarkers for cardiovascular diseases. Therefore, there is a lack of data regarding EVs that are produced locally in skeletal muscles. Moreover, whether the cargo of these EVs is modulated in order to improve the angiogenic response in the setting of tissue ischemia is currently unknown. Defining miR expression profile and modulation by ischemia might be especially important in the case of EVs, whose angiogenic activities could potentially be harnessed to develop novel therapies aimed at improving neovascularization and reducing tissue damage in patients with PAD.

miRs are increasingly recognized as important regulators of several biological and cellular processes such as growth, apoptosis, inflammation, metabolic activity and angiogenesis [9,10,11]. However, their specific role in the response to tissue ischemia in the context of vascular diseases is still poorly defined. We used a well-described mouse model of hindlimb ischemia [34] and next generation sequencing (NGS) to perform a global and unbiased evaluation of miR expression profile in skeletal muscles. Our results demonstrate that several miRs with potential angiogenic activities (angiomiRs) are significantly increased in ischemic skeletal muscles including Let-7b-5p, miR-148a-3p, miR-19b-3p and miR-25-3p. Let-7b-5p, which is also highly expressed in pericardial fluid exosomes, was previously shown to promote angiogenesis in vitro and in vivo through targeting of the antiangiogenic TGFBR1 [24]. In the context of cancer, Let-7b-5p was also shown to target SOCS1 [35], the inhibition of which is associated with a pro-angiogenic switch [36]. Also in the context of cancer, exosomal miR-148a-3p was shown to promote tumor angiogenesis through activation of the EGFR/MAPK signaling pathway by ERRFI1 inhibition [25]. Although the effect of miR-19b-3p on angiogenesis has not been directly studied, members of this family of miRs can stimulate the pro-angiogenic PI3K/AKT/mTOR pathway through targeted inhibition of PTEN [26]. Finally, miR-25-3p has been shown to promote endothelial cell angiogenesis in aging mice via targeting of TULA-2, leading to increased VEGFR-2 phosphorylation [27].

Our results therefore suggest that a group of angiomiRs are induced in skeletal muscles in response to ischemia. miRs can act locally in producing cells but can also be important mediators of paracrine signals via EVs that are involved in the communication between cells. This mechanism is especially relevant in the context of angiogenesis, where angiogenic signals need to be communicated to endothelial cells. Accordingly, the expression profile of miRs could be even more pertinent in EVs (vs. whole skeletal muscles) in order to provide insights on the angiogenic signals that can be transmitted to endothelial cells locally. Our NGS analysis indicates that a similar number of miRs can be detected in whole skeletal muscles (370), MVs (355) and exosomes (354). This is consistent with the fact that the expression profile of EV-derived miRs is similar to that of cells from which they originate [37]. However, in the present study we uncovered a distinctive set of angiomiRs that are upregulated and/or selectively packaged in EVs in the setting of tissue ischemia. For instance, miR-146b-5p and miR-152-3p are significantly increased by ischemia in MVs, whereas miR-150-5p is enriched in MVs when compared to whole ischemic skeletal muscles. We have previously shown that miR-146b-5p improves angiogenesis and ischemia-induced neovascularization through reduction of TRAF6-dependent inflammation [14]. miR-152-3p was shown to be crucial to maintain the angiogenic ability of endothelial cells through direct targeting of mesenchyme homeobox 2 (MEOX2) [28], whereas miR-150 targets SRC kinase signaling inhibitor 1 (SRCIN1), an important regulator of Src activity, and increases angiogenesis and neovascularization in atherosclerotic conditions [13]. In exosomes, miR-142a-3p and miR-21a-5p are significantly increased by ischemia, whereas miR-378-5p is enriched compared to ischemic muscles. miR-142a-3p was previously shown to modulate VEGF and angiogenesis in vitro and in vivo [29,38]. In a rat diabetic retinopathy model, miR-21a-5p was shown to activate the PI3K/Akt/VEGF signaling pathway and promote angiogenesis via repression of *PTEN* expression [30]. miR-378-5p can directly target Sufu, a negative regulator of the hedgehog (HH) pathway, which leads to activation of VEGF-dependant angiogenesis [33]. Interestingly, we identified two miRs that are enriched by ischemia both in MVs and exosomes: miR-486b-5p and Let-7e-5p. In a limb ischemia model, miR-486b-5p was previously shown to target PTEN, activating the AKT/MTOR/HIF-1α pathway and increasing angiogenesis [31]. Let-7e-5p was shown to enhance angiogenesis in endothelial cells through targeting of thrombospondin 1 (TSP1), a natural inhibitor of angiogenesis [32].

Collectively our results suggest that upon skeletal muscle ischemia, a group of miRs with pro-angiogenic activities are selectively packaged to be exported in EVs. The mechanisms of miR sorting into EVs are complex and involve several miR-binding proteins including heterogeneous nuclear ribonucleoproteins (e.g., hnRNPA2B1) and argonaute 2 (Ago2) that can bind specific miRs and shuttle them into EVs [39]. In the case of MVs, membrane proteins such as Caveolin-1 (Cav-1) are also involved [40]. Selective packaging of miRs has been described in several conditions including lung diseases, immune responses, neuroinflammation, cancer, diabetes and cardiovascular diseases [40]. However, the precise mechanisms that are responsible for EV packaging of specific miRs in each condition remain to be determined. In addition, the global functional action of the packaged miRs within EVs is also complex, considering that each miR can have multiple targets. Interestingly, it has been suggested that cells can generate a common functional signal through selective EV-mediated export of an overlapping set of miRs [41]. In the current study, we used bioinformatic approaches to perform prediction of functional miR targets and physiological/pathological functions that could be modulated by the group of miRs identified to be enriched in skeletal muscle EVs following ischemia. Our results confirm that as a group, these miRs are predicted to modulate several pathways that are involve in angiogenesis and neovascularisation including VEGF, MAPK, cGMP/PKG and PI3K/Akt [6] (Figure 6 and Figure 7).

Our study has important limitations. Although it provides a complete profiling of miR expression in skeletal muscles and EVs, we did not confirm by a second method (e.g., qPCR) all the miRs that were found to be significantly modulated by next generation sequencing (NGS). Therefore, the present report should be seen as exploratory, and future studies are needed to confirm the specific angiomiRs that are induced and transported by EVs in different ischemic conditions. In addition, we did not quantify the expression levels of target proteins and the effect of ischemia in skeletal muscles. The targets we describe for each potential angiomiR have been validated, often in the context of ischemia and angiogenesis, but not necessarily in the same animal model of hindlimb ischemia. Our study focuses on EVs and miRs that are produced locally in skeletal muscles. It has been proposed that angiogenic miRs contained in EVs present in the plasma could also contribute to protect against hindlimb ischemia [42]. The relative contribution of local (i.e., skeletal muscle) vs. peripheral blood EVs to ischemia-induced angiogenesis and neovascularisation remains to be determined.

In conclusion, in a model of peripheral artery diseases, our study demonstrates that ischemia is associated with a selective upregulation of miRs with pro-angiogenic activities (angiomiRs) in EVs that are produced locally in skeletal muscles. We found that certain angiomiRs are globally induced in EVs of different sizes, whereas other angiomiRs are specifically induced in MVs or exosomes. Collectively, the angiomiRs that we identified are expected to modulate several crucial pathways that are involved in angiogenesis and neovascularization. Our findings constitute a solid foundation for the identification of angiomiRs that could be targeted in order to improve EV angiogenic function. In novel therapeutic strategies, these reprogrammed EVs could be used as potent angiogenic vectors to improve neovascularization and potentially reduce tissue damage in patients with severe ischemic vascular diseases.

## Data Availability

The original contributions presented in the study are included in the article. Further inquiries can be directed to the corresponding author.
